# In Situ Melanoma of the Nipple and Areola: A Dermoscopic Report in Two New Cases

**DOI:** 10.5826/dpc.1102a01

**Published:** 2021-03-08

**Authors:** Giulio Tosti, Camilla Salvini, Alessia Barisani, Sabina Vaccari

**Affiliations:** 1 Divisione di Chirurgia del Melanoma, Sarcoma e Tumori Rari, IRCCS, Istituto Europeo di Oncologia, Milan, Italy; 2 Unit of Dermatology, USL Toscana Centro-Prato Hospital, Prato, Italy; 3 Dermatology, Department of Experimental, Diagnostic and Specialty Medicine, Policlinico Sant’Orsola-Malpighi, University of Bologna, Italy

**Keywords:** nipple, areola, melanoma, dermoscopy

## Introduction

The diagnosis of nipple and areola complex (NAC) lesions can be challenging even for expert dermatologists, since several differential diagnoses, including neoplastic and inflammatory conditions, should be acknowledged. NAC melanoma is rare; dermoscopy may aid in its correct evaluation. We report here 2 new cases of in situ NAC melanoma.

## Case Presentations

### Case 1

**Figure 1:**
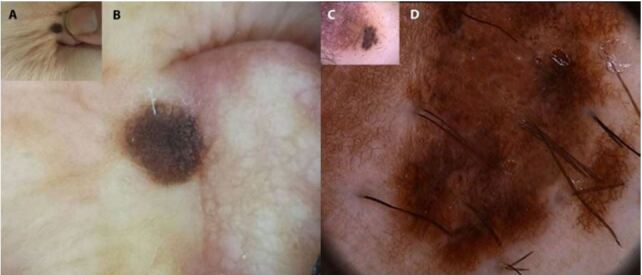
(A) Case 1. A 60-year-old Caucasian woman presenting an atypical dark pigmented lesion 4 mm in diameter located at the areola. (B) Dermoscopy showed a dark symmetrical macule characterized by a thickened atypical pigment network and irregular blotches (B, original magnification ×20). (C) Case 2. A 48-year-old man affected by an irregularly shaped macular lesion 7 mm in diameter at the left areola. (D) Dermoscopy shows light to darker brown pigmentation with a multi-component pattern and atypical pigment network (D, original magnification ×40).

A 60-year-old Caucasian woman presented with a 4 mm pigmented lesion on the areola at the edge of the nipple (Figure 1A). During the previous 4 months, the lesion had progressively darkened. Dermoscopy showed a dark symmetrical macule with a thickened atypical pigment network and irregular blotches (Figure 1B). The lesion was flat, with no signs of infiltration or discharge. Histopathology revealed an in situ melanoma.

### Case 2

A 48-year-old Caucasian man presented with a 7 mm irregularly shaped macule affecting his left areola (Figure 1C). He was asymptomatic and had a 100+ nevi body count. Dermoscopy showed light to darker brown pigmentation with a multi-component pattern and an atypical pigment network (Figure 1D). Also in this case histopathology revealed an in situ melanoma.

Both patients underwent wide excision with a 5-mm margin.

## Conclusions

NAC melanoma represents an uncommon diagnosis, and until now only a few cases have been reported in the literature. The first dermoscopic observation was reported by Shiga et al [1], who described a well-circumscribed, blackish macule (10 mm in size) on the left NAC; dermoscopy displayed pigment network, irregular blotches and blue-white veil on the normal-colored NAC; and histopathology revealed a 0.3-mm melanoma. Later, Cinotti et al [2] reported another case of melanoma of the areola showing, on dermoscopy, irregular pigmented network with blotches and blue-white veil.

The dermoscopic features of our 2 cases are concordant with those previously described. The differential diagnosis includes: melanocytic nevi, melanosis, and pigmented mammary Paget disease (MPD).

Melanocytic nevi in this site often represent a challenging diagnosis, partly because of the irregular surface of the NAC, with duct orifices modifying the distribution of the pigment, thus favoring false-positive diagnoses. In a multicentric study, 16 out of the 66 nevi evaluated on dermoscopy (24%) were classified as melanoma using the 7-point checklist [2]. Moreover, the normal areola can display a brown pigment network and might sometimes be a site of friction and repetitive trauma.

Melanosis of the NAC can share some clinical features with melanoma, representing a possible cause of diagnostic concern. Melanosis may reveal a network and a cobblestone pattern which may be difficult to differentiate from melanocytic lesions.

Dermoscopic features of pigmented MPD include structureless brown and red areas, pinkish background, irregular vessels and irregular blue and brown dots.

Only a few cases of NAC melanoma with dermoscopic findings were reported in the literature. The cases presented here represent a clinico-dermoscopic report of in situ NAC melanoma.
